# Evaluating project management office adoption for value-oriented governance in large-scale construction projects

**DOI:** 10.1038/s41598-026-44726-8

**Published:** 2026-05-05

**Authors:** Ehab A. Mlybari, Mohamed T. Elnabwy

**Affiliations:** 1 Department of Civil Engineering, College of Engineering and Architecture, Umm Al-Qura University, Makkah, Saudi Arabia; 2 School of Architecture and Built Environment, Northumbria University, Newcastle upon Tyne, NE1 8ST UK

**Keywords:** Project Management Office (PMO), Structural Equation Modeling (SEM), Change Management, Risk Management, Organizational Value, Haramain Expansion, Business and management, Business and management, Environmental social sciences

## Abstract

Although Project Management Offices (PMOs) are increasingly recognized as mechanisms for improving coordination, governance, and organizational value realization across projects, empirical understanding of their adoption in the construction sector remains limited. Existing studies have largely emphasized PMO typologies and maturity levels, offering little quantitative evidence on how internal organizational capabilities and external environmental factors jointly influence adoption outcomes and value-oriented governance through effective PMO adoption. Addressing this gap, this study develops and validates a multi-factor structural model to evaluate PMO adoption (PMOA) in large-scale construction settings. Using the Haramain Expansion Project in Saudi Arabia as an empirical case, data were collected from 60 professionals across government, semi-government, and private organizations. Partial Least Squares Structural Equation Modelling (PLS-SEM) was employed to assess relationships among four latent constructs including Knowledge and Awareness (KAF), Implementation Excellence (IEF), Change Management (CMF) and External and Risk Management (ERMF). The model exhibited strong reliability and predictive accuracy, explaining 97.6% of the variance in PMOA (R² = 0.976). Implementation Excellence (β = 0.366) and Change Management (β = 0.349) emerged as the strongest determinants, followed by ERMF (β = 0.271) and KAF (β = 0.250). These findings provide the first empirical validation of a multi-dimensional PMO adoption framework in the Middle Eastern construction context, advancing understanding of how capability integration, risk preparedness, and governance mechanisms collectively support sustained organizational value and resilience in complex infrastructure environments.

## Introduction

The construction industry operates in a dynamic and complex environment, often marked by high levels of uncertainty, multidisciplinary coordination, and interdependent activities. Each construction project presents distinct goals, risks, and constraints, all of which significantly influence organizational performance^1,2^. While project-specific decision-support systems are common, the growing complexity of project portfolios has led to a shift in focus toward centralized, organization-wide project governance mechanisms. One such mechanism is the Project Management Office (PMO), which is increasingly recognized as a strategic function that supports consistency, efficiency, and alignment across projects^3–5^.

The PMO is designed to enhance organizational capability in managing multiple projects by standardizing processes, improving resource allocation, facilitating knowledge transfer, and ensuring alignment with strategic goals. In the construction sector, PMOs play a pivotal role in driving implementation excellence, managing change, addressing external risks, and embedding a culture of continuous improvement^6^. As such, the effective adoption of PMOs is increasingly viewed as a critical success factor for achieving sustained performance at both the project and organizational levels.

Despite these recognized benefits, many construction firms underutilize PMOs or implement them in a fragmented, administrative capacity, rather than as strategic enablers^7^. This limited view often stems from a lack of awareness, unclear implementation pathways, or insufficient adaptation to the unique challenges of the construction sector^8^. As a result, there remains a significant gap in understanding the key drivers that influence PMO Adoption (PMOA) and how they interact to shape success.

In this study, PMOA is defined as the extent to which PMO structures, governance mechanisms, and standardized practices are formally institutionalized and operationalized within an organization’s project environment. PMOA is conceptually distinguished from PMO maturity and PMO performance. While PMO maturity reflects the developmental progression of PMO capabilities and PMO performance refers to achieved efficiency or outcome improvements, PMOA focuses specifically on the successful establishment, integration, and functional embedding of the PMO within organizational project governance systems.

Unsuccessful PMO adoption may lead to fragmented governance arrangements, duplicated decision-making structures, weak coordination across projects, ineffective change control, and limited organizational learning^7^. In complex construction environments, particularly those involving large-scale or multi-stakeholder projects, such shortcomings can exacerbate schedule delays, cost overruns, and stakeholder conflicts^2^. Clarifying the conceptual boundaries and practical implications of PMOA is therefore essential for understanding not only whether PMOs exist within organizations, but whether they are meaningfully embedded and capable of fulfilling their intended governance and coordination roles.

While prior PMO research has provided valuable insights into PMO typologies, maturity models, and performance outcomes, much of this work has remained descriptive or focused on post-implementation effectiveness, offering limited quantitative insight into Project Management Office Adoption (PMOA) as a distinct organizational process^9^. Insufficient attention has been given to how internal organizational capabilities and external environmental and risk-related factors jointly influence the formal establishment, institutionalization, and functional embedding of PMOs within construction project governance systems. Addressing this gap, the present study develops and empirically validates a conceptual framework using Structural Equation Modelling (SEM) to examine the influence of four core PMO capability dimensions: Knowledge and Awareness (KAF), Implementation Excellence (IEF), Change Management (CMF), and External and Risk Management (ERMF). Drawing on survey data collected from diverse industry stakeholders, the study quantifies the interrelationships among these factors and advances theoretical understanding of PMOA while providing evidence-based guidance for enhancing PMO strategies in large-scale and complex construction environments.

## Literature review

### The strategic role of PMOs in construction

Despite the growing recognition of PMOs as strategic governance mechanisms, empirical evidence suggests that PMO adoption within the construction sector remains uneven and often fragmented^9^. Many construction organizations establish PMOs in response to external pressures or project scale rather than through deliberate institutionalization, resulting in limited authority, unclear mandates, and weak integration with organizational decision-making structures. Prior studies report that PMOs in construction frequently operate in administrative or reporting roles, with constrained influence on strategic planning, portfolio coordination, and performance control^10^. This disconnect between formal PMO existence and effective organizational embedding highlights that PMO adoption cannot be assumed based solely on maturity models or performance outcomes. Instead, PMO adoption represents a distinct organizational challenge shaped by internal capabilities, governance readiness, and the ability to respond to external and project-level uncertainty^10^.

Building on this perspective, prior studies indicate that PMO Adoption is influenced less by the formal existence of a PMO and more by the organizational capabilities that support its legitimacy, operational effectiveness, and long-term stability. In construction contexts, limitations in stakeholder understanding, inconsistent implementation practices, weak change management, and inadequate responses to external uncertainty have all been identified as key barriers to PMO institutionalization. These challenges provide the theoretical basis for examining Knowledge and Awareness, Implementation Excellence, Change Management, and External and Risk Management as capability-based antecedents of PMO Adoption.

### Knowledge and awareness (KAF)

Successful PMO adoption begins with stakeholder awareness and understanding of its value. Awareness encompasses both internal (staff and management) and external (clients, regulators) knowledge of PMO functions, objectives, and strategic benefits^11^ emphasizes the importance of PMO literacy as a precursor to effective implementation, while studies in developing regions reveal that lack of awareness remains a major barrier to PMOA^2^. Knowledge dissemination and capacity-building initiatives are therefore essential to foster buy-in and reduce resistance.

### Implementation excellence (IEF)

Implementation excellence reflects the degree to which organizations apply PMO principles, tools, and methodologies in a consistent and competent manner. Empirical research links high PMO adoption context to capabilities in methodology development, standardized processes, and strategic alignment^12^. According to Hobbs et al. ^13^, effective implementation frameworks—particularly those aligned with performance measurement tools like the Balanced Scorecard—are essential for embedding PMOs within organizations and ensuring they deliver tangible strategic benefits.

### Change management (CMF)

Change is inherent in construction projects, and PMOs are increasingly tasked with managing transitions in scope, leadership, and stakeholder expectations. Effective change management practices allow PMOs to absorb shocks such as design revisions or managerial turnover, without compromising performance^9,14^. Research shows that PMOs capable of managing adaptive cycles maintain stronger stakeholder confidence and organizational resilience^15^. CMF also includes cultivating a change-embracing culture, which is particularly important in risk-prone construction environments.

### External and risk management (ERMF)

External and risk management encompasses a PMO’s capability to anticipate and address influences outside its direct oversight—such as policy changes, economic volatility, or supplier uncertainty. According to Artto et al^16^., PMOs play a pivotal role in enhancing early-stage risk identification and aligning mitigation strategies with organizational goals. Furthermore, research by^17^indicates that organizations with mature PMO structures demonstrate greater resilience and continuity amid external shocks, positioning the PMO not just as a project enabler, but as a key component of strategic risk governance.

### The use of SEM in construction and PMO research

SEM has become an indispensable analytical tool in construction management research, particularly for examining complex relationships between latent constructs that cannot be directly observed. Its ability to simultaneously assess multiple pathways of influence makes it uniquely suited for investigating multifaceted organizational phenomena^18^. In construction contexts, SEM has been successfully applied to study technology adoption patterns^19,20^, risk management frameworks^21–23^, and organizational performance metrics^24^. Its applicability extends to PMO studies, where it enables researchers to simultaneously assess the influence of multiple drivers—such as awareness, process maturity, and environmental uncertainty—on PMO adoption or effectiveness^25^. The present study employs SEM to uncover the interdependencies among four core PMO-related constructs and their collective impact on PMOA, thereby contributing both methodological depth and empirical clarity to the field. To consolidate the insights drawn from prior research, Table 1 presents a structured summary of the main latent constructs, their associated sub-factors, empirical findings, and supporting references. These constructs form the theoretical backbone of this study’s proposed SEM, aimed at explaining the dynamics influencing PMOA in construction project environments. The categorization not only reflects key dimensions derived from the literature but also provides the basis for the hypotheses formulated in the next section.

Several concepts frequently discussed in prior PMO studies such as PMO support and buy-in, PMO performance, and PMO stability or credibility—are not treated in this study as independent outcome variables or separate predictors. Existing research consistently indicates that stakeholder support and organizational buy-in are central to the legitimacy and institutionalization of PMO structures^26,27^, while demonstrated PMO performance and perceived value reinforce organizational commitment and long-term sustainability^28^. In addition, PMO stability and credibility are widely linked to the durability of governance arrangements and continued organizational reliance on PMOs under dynamic conditions^14^. In this study, these elements are therefore conceptualized as supporting mechanisms and manifestations of successful PMO Adoption (PMOA), reflecting the extent to which PMO structures are accepted, operationalized, and sustained within organizational governance systems. Accordingly, Table 1 synthesizes these elements as supporting empirical evidence reported in prior literature, rather than as standalone hypotheses or parallel constructs.

**Fig. 1:**
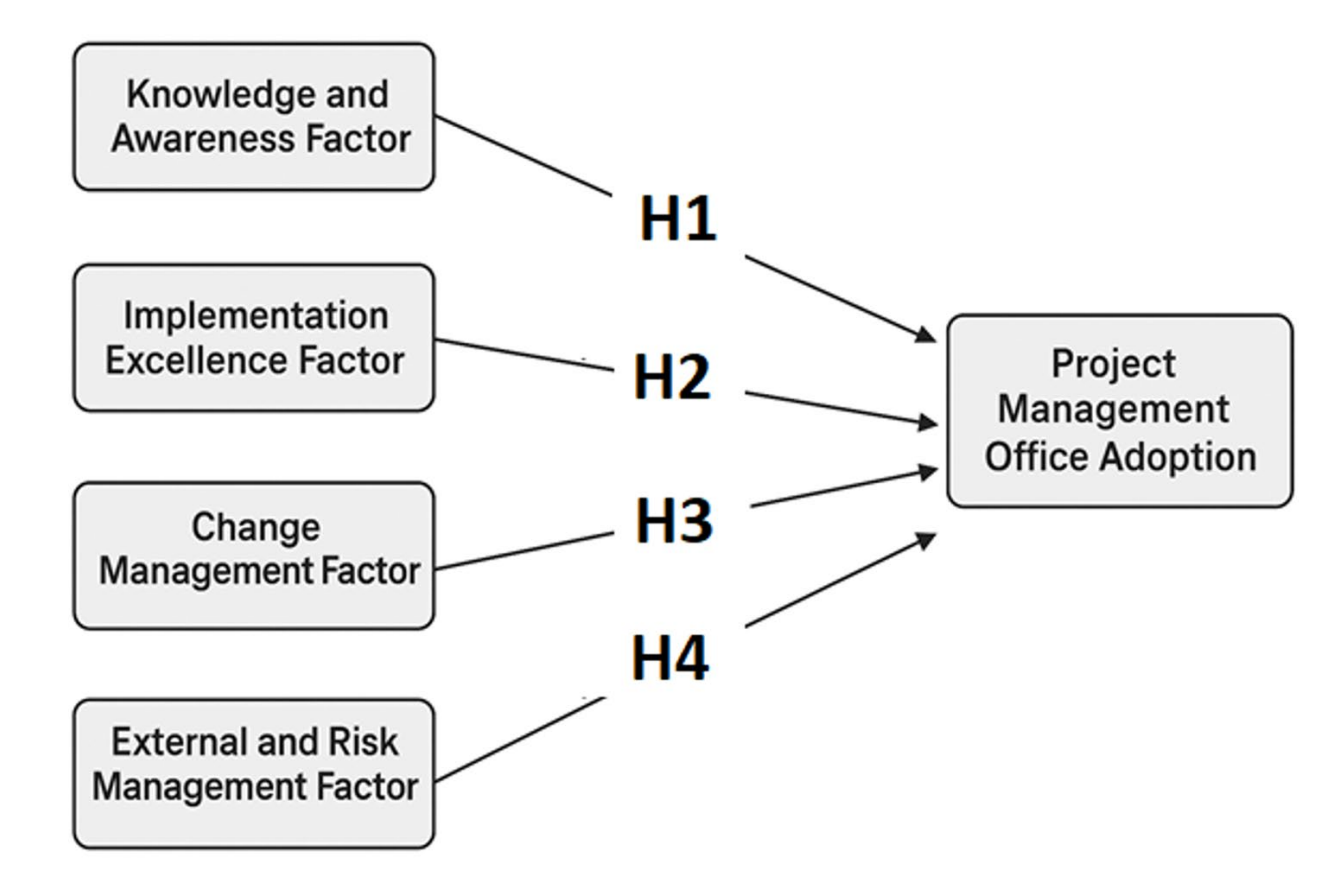
Conceptual framework for factors influencing PMO adoption in large-scale construction projects.

Based on the reviewed literature, this study proposes that PMO Adoption (PMOA) is influenced by a set of organizational and governance-related capabilities. KAF are expected to facilitate stakeholder understanding, acceptance and support for PMO structures, thereby enabling adoption (Fig. 1). IEF reflects the organization’s ability to consistently apply PMO methodologies and processes, which is essential for embedding PMO practices within project governance. CMF captures the organization’s capacity to adapt PMO structures to evolving project conditions and stakeholder requirements, supporting sustained adoption. ERMF represents the ability of the PMO to respond to external pressures and uncertainties, reinforcing its relevance and legitimacy within complex construction environments. Accordingly, the following hypotheses are proposed:

H1: Knowledge and Awareness (KAF) positively influence PMOA.

H2: Implementation Excellence (IEF) positively influences PMOA.

H3: Change Management (CMF) positively influences PMOA.

H4: External and Risk Management (ERMF) positively influence PMOA.

**Table 1:** Summary of Key Latent Variables, Sub-Factors, Main Findings, and References Supporting the PMOA Framework.

Main Factor	Sub-factor	Main findings	References
Knowledge and Awareness (KAF)	Internal awareness	Stakeholder knowledge and understanding facilitate acceptance of PMO roles and support the institutionalisation of PMO structures within organisations	^10^
	External visibility	Awareness of PMO purpose and value among external stakeholders supports organisational alignment and reinforces PMO adoption	^3,29^
	Perceived importance	Recognition of the strategic importance of PMOs encourages organisational commitment and supports formal PMO adoption	^27,30^
Implementation Excellence (IEF)	Application of PMO tools	Consistent application of PMO tools and practices enables the operationalisation and embedding of PMO functions within project governance	^12,28^
	Methodological consistency	Methodological consistency strengthens organisational readiness and supports sustained adoption of PMO practices	^12,28^
Change Management (CMF)	Design/scope flexibility	Adaptive capability in managing design and scope changes supports the integration and continuity of PMO structures under dynamic conditions	^9,13^
	Leadership adaptation	Leadership responsiveness to change facilitates acceptance and sustained adoption of PMO governance arrangements	^31,32^
External and Risk Management (ERMF)	Risk identification	Proactive risk identification enhances the relevance and legitimacy of PMOs, supporting their adoption in complex environments	^16,33^
	Response to external disruptions	Effective response to external disruptions reinforces the role of PMOs as governance mechanisms and supports continued adoption	^17,34^

## Research methodology

**Fig. 2:**
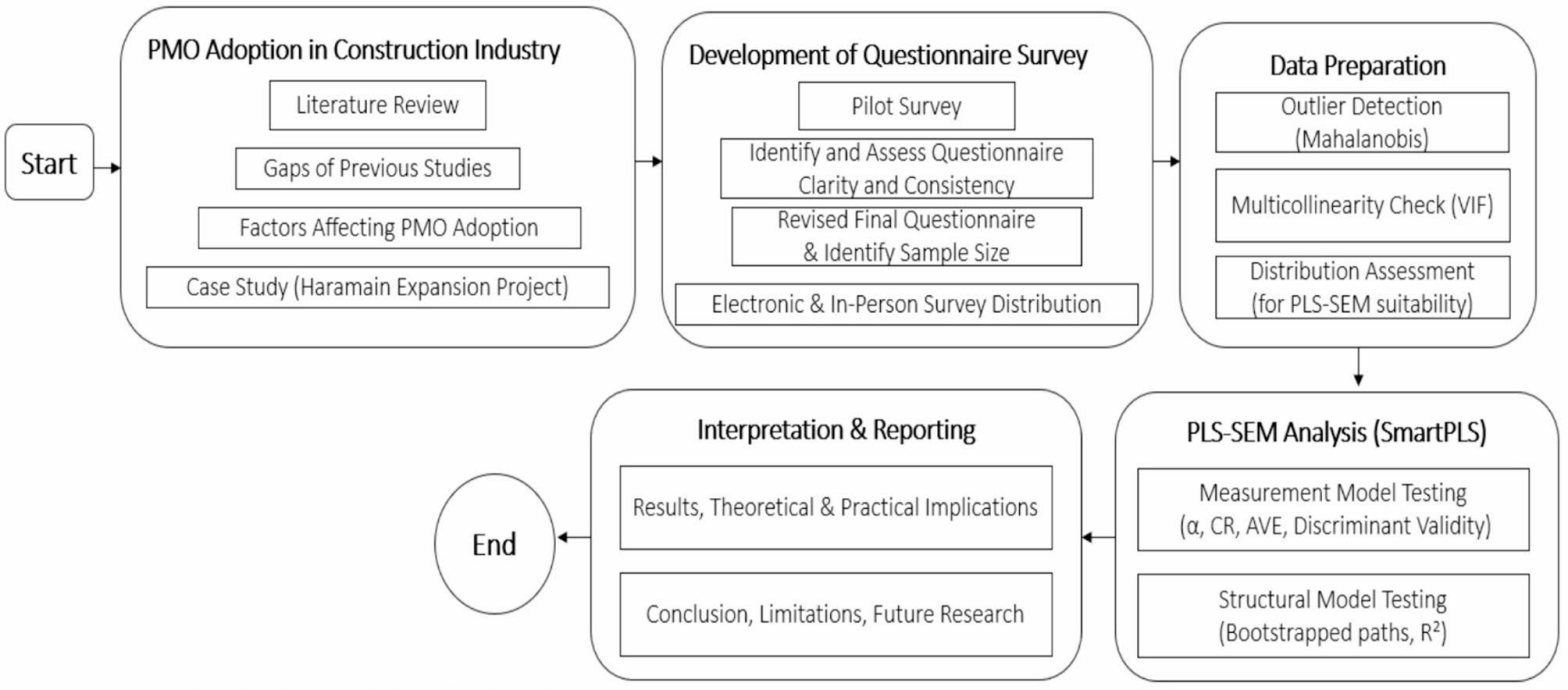
Methodology framework.

This section outlines the empirical research design adopted to examine the determinants of PMO Adoption (PMOA) in large-scale construction projects. The study employs a quantitative approach and applies PLS-SEM to test the proposed structural relationships among the identified constructs. Figure 2 presents the sequential flow of the research process. The study begins with a structured literature review and contextual framing of the empirical case, followed by questionnaire development, pilot testing, and survey distribution. Subsequent stages involve systematic data preparation procedures, including outlier detection, multicollinearity assessment, and suitability checks for PLS-SEM, culminating in model estimation using SmartPLS. The final stage focuses on interpreting results and deriving theoretical and practical implications. Each phase of the research process was designed to ensure methodological rigor, internal consistency, and contextual validity.

### Theoretical grounding of the research design

The research design is explicitly grounded in organizational capability theory and project governance theory, which jointly inform the conceptualization of PMO Adoption (PMOA) and the hypothesized structural relationships examined in this study. From an organizational capability perspective, sustained governance structures emerge from an organization’s ability to integrate and deploy internal competencies under conditions of complexity and uncertainty. In this study, KAF, IEF, CMF, and ERMF are conceptualized as capability-based dimensions representing distinct organizational routines, managerial processes and adaptive capacities. PMO adoption is therefore treated as an institutional outcome emerging from the combined influence of these capabilities rather than as a static structural attribute. Project governance theory further frames the PMO as a formal governance mechanism responsible for coordination, oversight, control, and strategic alignment across multiple projects and stakeholders. Within this perspective, successful PMO adoption reflects the degree to which governance arrangements are formally institutionalized and embedded within the organizational project environment. The structural paths tested in this study therefore represent theoretically derived linkages between governance-relevant organizational capabilities and the institutional integration of PMO structures. By grounding the research design in these complementary theoretical perspectives, the study ensures coherence between the conceptual framework, hypothesis development, measurement specification, and interpretation of results.

### Literature review & case study context

A comprehensive literature review served as the initial data-gathering stage of this research. Engagement with relevant academic studies and prior empirical work provided a strong theoretical foundation, helped avoid duplication of existing efforts, and informed the development of the survey instrument. The review focused on PMO adoption, governance mechanisms, organizational capabilities, and the application of structural equation modelling in construction and project-based environments.

The empirical investigation was conducted within the context of the Haramain Expansion Project in Saudi Arabia, a large-scale, strategically significant, government-led construction program. The project is characterized by high organizational complexity, the involvement of multiple public and private stakeholders, and extensive coordination requirements across numerous work packages. Governance and oversight are largely centralized within public-sector institutions, supported by formal PMO structures responsible for planning, execution, and performance monitoring across organizational interfaces.

The scale, governance intensity, and multi-stakeholder nature of the Haramain Expansion Project make it a suitable empirical context for examining PMOA) in complex construction environments. The project operates under formal governance arrangements in which PMO structures were established to support coordination, reporting, and oversight across multiple implementing entities. These characteristics provide a realistic setting for testing the proposed SEM model and for empirically investigating not merely the presence of a PMO, but the extent to which organizational capabilities and governance-related factors influence the formal institutionalization and embedding of PMO structures within large construction programs.

### Research sample

This study employed purposive sampling to target professionals directly involved in PMO-related governance, decision-making, and operational implementation within the Haramain Expansion Project. The targeted participants included client-side representatives, consultants, and contractor personnel occupying roles related to project controls, planning, reporting, and portfolio or program management. The population, approximately 150 professionals, included individuals from government, semi-government, and private sectors.

Respondents from government and semi-government entities primarily represent client-side and owner-side roles within the project governance structure. Their involvement relates mainly to strategic decision-making, definition of PMO mandates, governance oversight, coordination across stakeholders, and monitoring of PMO-related adoption at the portfolio and program levels. In contrast, respondents from private-sector organizations primarily represent delivery-side roles, including project execution, coordination, and the operational implementation of PMO practices.

The dominance of government and semi-government respondents reflects the ownership and governance structure of the investigated project. The Haramain Expansion Project is a large-scale, government-owned construction program, where PMO-related authority, decision-making, and oversight responsibilities are largely centralized within public-sector institutions. Accordingly, the sample composition and size are consistent with the project context and the distribution of PMO-related roles across participating organizations.

Using Cochran’s formula with finite population correction, assuming a 95% confidence level, a 5% margin of error, and a conservative population proportion of 50%, a minimum sample size of 53 responses was required based on the size of the target population. A total of 60 complete responses were collected, exceeding the minimum requirement and providing sufficient statistical power for the analysis. Respondents reflected a variety of educational backgrounds, including PhD, Master’s and Bachelor’s.

### Pilot study & instrument validation

The pilot study involved five participants comprising PMO experts and project management professionals directly engaged in the Haramain Expansion Project. The pilot phase was conducted to assess item clarity, contextual relevance, and construct coverage prior to large-scale data collection. Given its limited size, the pilot study was not intended for statistical testing but to support content and face validity of the measurement instrument.

The pilot study was designed to assess item clarity, contextual relevance, and construct coverage rather than to perform statistical validation. Given its limited size and project-specific focus, the pilot phase primarily supported content validity and face validity. Potential limitations related to context specificity were subsequently addressed through rigorous measurement model assessment, including reliability, convergent validity, and discriminant validity testing using PLS-SEM in the full sample.

### Data collection & ethical considerations

The finalized questionnaire was distributed electronically and through in-person channels to purposively selected respondents. Participation was entirely voluntary, and informed consent was obtained prior to survey completion. The study was conducted in full compliance with institutional, national, and international ethical standards governing research involving human participants. All procedures followed the Declaration of Helsinki and the ethical guidelines of Umm Al-Qura University (Makkah, Saudi Arabia), with approval granted by the College of Engineering Research Committee. Participants were briefed on the study’s objectives, procedures, and their rights, including the freedom to withdraw at any time without penalty. To ensure data integrity and confidentiality, no personally identifiable information was collected, and all responses were anonymized and used solely for academic research. Data handling complied with institutional and national data-protection regulations.

### Construct measurement

The measurement instrument used in this study was developed based on an extensive review of prior empirical research on the PMO, organizational capabilities, and project governance. Measurement items were adapted from established studies and tailored to the context of large-scale construction projects to ensure relevance and clarity. To enhance content validity and contextual appropriateness, the initial set of items was reviewed and refined through a pilot study involving PMO experts and project management professionals engaged in the Haramain Expansion Project. Feedback from the pilot study resulted in minor refinements to item wording and structure, leading to the final questionnaire used for data collection. All measurement items were assessed using a five-point Likert scale.

The four exogenous constructs in the model were operationalized as latent variables measured reflectively through multiple indicators capturing organizational perceptions and capability related practices. KAF was measured using indicators reflecting stakeholder understanding of PMO roles, governance authority, and perceived strategic value. IEF was measured through indicators capturing the consistent application of PMO tools, methodologies, and standardized project management practices. CMF was operationalized using indicators related to organizational adaptability in managing scope changes, leadership transitions, and stakeholder alignment. ERMF was measured using indicators associated with risk identification, responsiveness to external disruptions, and the management of environmental uncertainty. These indicators collectively represent the organizational capability dimensions most frequently identified in prior PMO and construction management literature.

PMO adoption was conceptualized as an endogenous latent outcome construct representing the overall level of institutionalization, governance integration, and functional embedding of PMO structures within the organizational project environment. In line with the study objective, PMO adoption was not measured using a separate set of survey indicators. Instead, its level was inferred through its structural relationships with the four organizational capability constructs included in the model, namely KAF, IEF, CMF, and ERMF. This approach reflects the conceptualization of PMO adoption as an organizational process emerging from the combined influence of internal capabilities and governance related practices rather than as a directly observed or independently measured variable.

Accordingly, the assessment of PMO adoption focuses on the explanatory power of the structural model and the statistical significance of the relationships between the antecedent constructs and PMO adoption. Construct validity and reliability were therefore evaluated for the exogenous capability constructs at the measurement model level, while PMO adoption was evaluated at the structural level using path coefficients and the coefficient of determination. This measurement specification is consistent with established applications of SEM in construction management and organizational research and ensures alignment between the conceptual framework, empirical analysis and interpretation of results.

### Data preparation

Post-collection, all 60 survey responses were complete and retained for analysis. Numeric coding was applied, and data cleansing included outlier detection via Mahalanobis distance and multicollinearity checks using Variance Inflation Factor (VIF), which confirmed no significant issues. Although Smart PLS-SEM is robust to deviations from normality, descriptive analysis confirmed that the data distribution did not violate extreme non-normality.

### Data analysis

A variance-based PLS-SEM approach was selected to analyze the proposed model due to its suitability for theory testing involving multiple latent constructs and complex causal relationships. PLS-SEM is particularly appropriate for studies aiming to explain and predict key outcome variables, such PMOA, in organizational settings characterized by moderate sample sizes and non-normal data distributions, which are common in construction management research. Hair et al^18^. recommend PLS-SEM for exploratory, prediction-driven studies over CB-SEM^35^. specifically highlight Smart PLS’s advantages in maximizing explained variance and its capability to model formative constructs Smart PLS also supports bootstrap testing of path coefficients, enabling robust hypothesis testing even with small, non-normal samples. The analysis will be conducted in two phases. In the measurement model stage, we will evaluate internal consistency (Cronbach’s alpha, Composite Reliability), convergent validity (Average Variance Extracted), and discriminant validity (Fornell-Larcker and HTMT). The structural model stage will assess path coefficient significance through bootstrapping, coefficient of determination and (R²). This comprehensive two-phase approach ensures both the reliability of constructs and the explanatory power of relationships within the PMO adoption framework. Finally, the study concludes with interpretation, reporting of results, and implications for theory, practice, and future research.

Although data were collected from a single mega-project, this study does not adopt a descriptive case-study approach. Instead, the Haramain Expansion Project is used as an analytical empirical context to test a theoretically grounded structural equation model of PMO adoption. This approach allows the examination of hypothesized relationships among latent constructs within a real-world governance setting while acknowledging contextual limitations.

## Results and discussion

### Descriptive statistics and respondent profile

A total of sixty completed responses were collected from professionals directly involved in the Haramain Expansion Project, offering a strong foundation for this study’s empirical insights. The respondent pool was dominated by government agency participants, representing 81% of the sample and including individuals from key institutions like the General Presidency for the Affairs of the Two Holy Mosques and Makkah Municipality as outlined in Table 2. Semi-government entities comprised around 7%, while private-sector participants accounted for approximately 12%. Functionally, respondents were evenly distributed across roles, with many identifying as project owners, consulting professionals, contractors, or holding other relevant responsibilities; this diversity in organizational activity ensures that technical, managerial, and operational perspectives are well represented.

Regarding PMO presence within their organizations, a notable majority of 83% confirmed that their entities had an established PMO, with oversight over project operations, while the remaining 17% indicated no PMO structure. This prevalence of PMOs reinforces the appropriateness of analyzing latent variables related to PMO adoption within this context.

The educational qualifications of the respondents were robust: around 65% held bachelor’s degrees, 25% had master’s qualifications, and approximately 3% held doctoral degrees. Professional experience among participants was equally varied, encompassing those with less than five years of sector involvement, those with five to twenty years, and seasoned professionals with over twenty years in the construction industry. This breadth of educational attainment and work experience introduces a valuable interplay of theoretical knowledge and practical insight.

The profile of this sample reveals significant implications for the study. The dominance of government agency participation highlights the key role of public-sector leadership in PMO-related decisions within the Haramain project. The widespread incidence of PMO structures suggests an organizational readiness that enhances the relevance of our model for investigating PMO adoption. Moreover, participation from consulting and contracting firms contributes a layered perspective across different stages of project implementation. Lastly, the wide range of educational and experiential backgrounds enriches the findings by drawing from both emerging professionals and seasoned experts, thereby supporting the credibility, depth, and reliability of the results section that follows.

While organizational affiliation and PMO presence were captured at an aggregate level to characterize the sample, further disaggregation by organization type was not pursued, as the primary focus of the analysis was on testing the structural relationships between latent constructs and PMOA.

**Table 2:** Summary of respondent demographics and organizational profile.

Category	Classification	Frequency	Percentage
Organization Type	Government	49	81.70%
	Semi-Government	4	6.70%
	Private	7	11.60%
Organization Activity	Owner	26	43.30%
	Contracting	8	13.30%
	Consulting	13	21.70%
	Others	13	21.70%
Availability of PMO	Exist	50	83.30%
	Not Exist	10	16.70%
Education Level	Bachelor’s Degree	43	71.70%
	Master’s Degree	15	25.00%
	Ph.D.	2	3.30%
Years of Experience	Less than 5 Years	26	43.30%
	5–20 Years	23	38.30%
	More than 20 Years	11	18.40%

### PMO awareness, need, and applicability

In this study, references to the state of the PMO relate to its adoption context and organizational positioning rather than to performance or maturity outcomes. Accordingly, the analysis in this section focuses on descriptive indicators that characterize PMO presence and governance context, while performance-related evaluation falls outside the scope of the present investigation.

The analysis of professional awareness concerning PMO functions within the Haramain Expansion Project reveals mixed levels of familiarity. While 40% of participants demonstrated strong knowledge of PMO fundamentals such as their general concept and benefits—fewer showed familiarity with specific dimensions: PMO types (32%), roles (35%), structural components (30%), and establishment steps (27%). The least understood aspect was PMO maturity models, with 45% expressing very low awareness. Interestingly, when self-assessing overall PMO knowledge, 27% rated it very high, 38% high, and only 10% very low. This mismatch suggests that strategic recognition of PMOs remains more prevalent than detailed operational understanding (Table 3).

These results align with external findings. The State of the PMO 2010 report by PM Solutions indicates that mature PMOs correlate with improved performance, reporting a 31% reduction in failed projects and 30% of projects finishing under budget. A comprehensive review also underscores PMOs’ pivotal role in knowledge management—centrally organizing lessons learned and enabling improved decision-making. Furthermore, contemporary analyses emphasize that raising PMO visibility through executive sponsorship, process standardization, and clear role definition significantly enhances their effectiveness^17^.

Perceived necessity of PMOs in the Haramain project is high. Among public-sector respondents, 72% emphasized PMO integration as essential, compared to just 4% who disagreed. In the private sector, 46% saw a strong value in PMOs, while only 8% did not. This broad support indicates a growing consensus across organizational types that PMOs are integral to governance. Regarding practical applicability, 52% of professionals identified PMOs as perceived importance for PMO adoption for managing risks and overcoming project challenges. Nearly half believed PMOs facilitate stakeholder coordination (47%), control budgets more effectively (48%), and provide valuable technical support (40%). These perspectives are supported by PM Solutions’ findings that PMOs contribute notably to risk mitigation, quality assurance, and strategic project alignment. Overall, although in-depth technical knowledge about PMOs remains limited among some participants, there is clear acknowledgment of their strategic value and practical utility. This gap between recognition and understanding highlights the need for targeted organizational development initiatives such as formal training and knowledge-sharing practices to ensure that PMO awareness translates into effective implementation and capability.

**Table 3:** Summary of PMO awareness, perceived need, and applicability.

Dimension	Indicator	High/positive response	Low/negative response
Awareness	General PMO Concept & Benefits	40%	–
	PMO Types	32%	–
	PMO Roles	35%	–
	PMO Structure	30%	–
	Steps to Establish a PMO	27%	–
	PMO Maturity Awareness	–	45%
Overall self-assessment	Very High PMO Knowledge	27%	–
	High PMO Knowledge	38%	–
	Very Low PMO Knowledge	–	10%
Perceived need	Need for PMO in Government Projects	72%	4%
	Need for PMO in Private Sector Projects	46%	8%
Applicability	Coordination Among Stakeholders	47%	3%
	Resolving Risks and Challenges	52%	–
	Supporting Budget Control	48%	–
	Delivering Technical Support	40%	–

### Measurement model assessment

The measurement model was evaluated to ensure the reliability and validity of all constructs included in the study. The assessment followed established PLS SEM guidelines and covered indicator reliability internal consistency reliability convergent validity discriminant validity and multicollinearity. The revised results after indicator refinement are summarised in Tables 4, 5, 6 and 7.

#### Indicator reliability

**Fig. 3:**
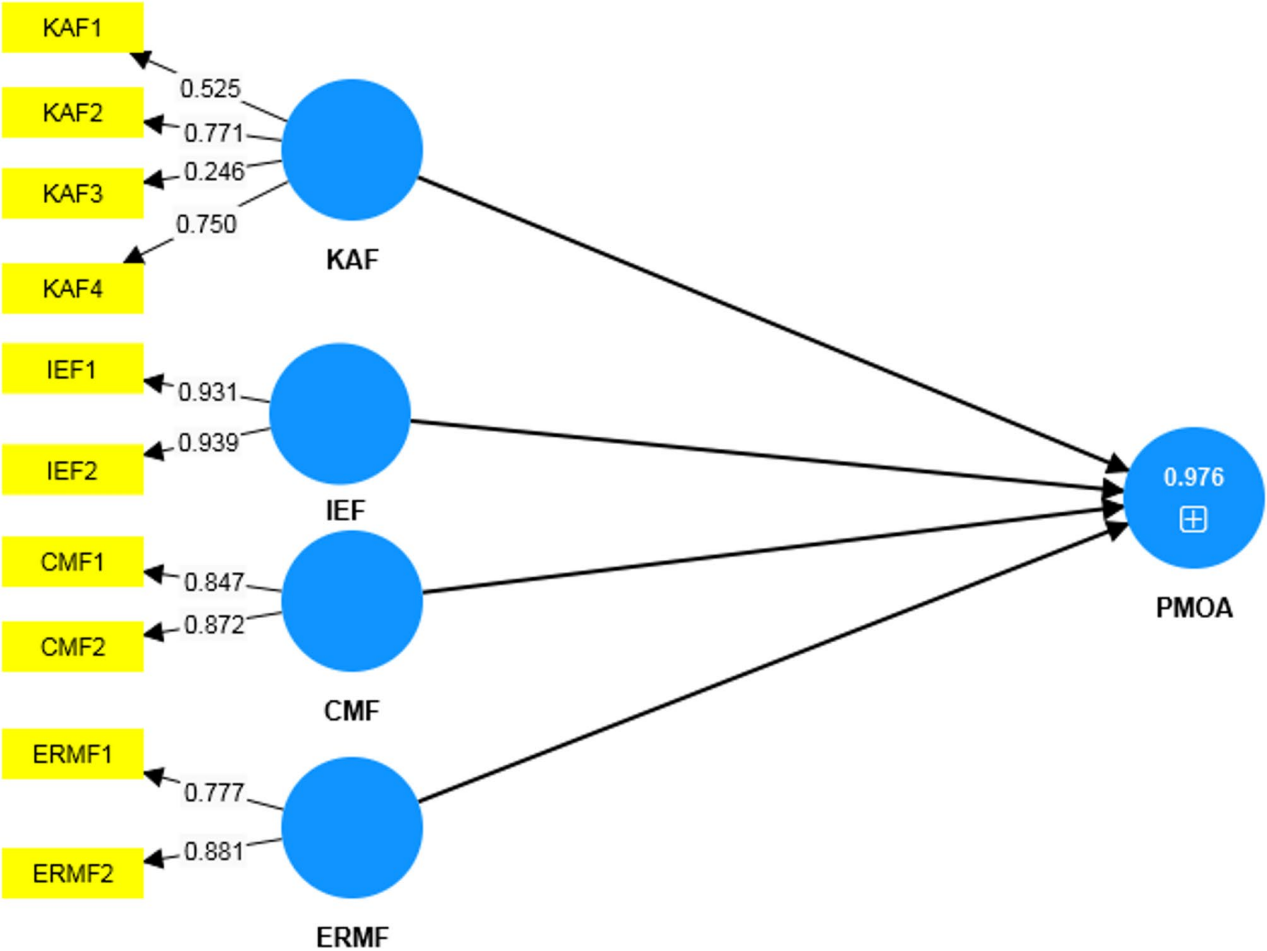
Measurement model PLS-SEM (outer loading values).

Indicator reliability was examined using outer loadings as illustrated in Fig. 3 and summarised in Table 4. In line with established guidelines outer loadings above 0.70 are preferred while values above 0.60 may be accepted in applied and exploratory research contexts. The revised measurement model demonstrates that most indicators exhibit satisfactory loadings. The Implementation Excellence construct shows strong indicator reliability with IEF1 and IEF2 loading at 0.931 and 0.939 respectively as presented in Fig. 3; Table 4. Similarly, the Change Management construct demonstrates robust loadings with CMF1 at 0.847 and CMF2 at 0.872. Indicators associated with External and Risk Management also exhibit acceptable reliability with ERMF1 loading at 0.777 and ERMF2 at 0.881. For the Knowledge and Awareness construct one indicator KAF3 exhibited a very weak loading in the initial estimation and was therefore removed from the model. Following this refinement the remaining indicators demonstrate improved loadings with KAF1 at 0.628 KAF2 at 0.785 and KAF4 at 0.762 as reported in Table 4 and illustrated in Fig. 3. This refinement enhanced the overall quality of measurement for the Knowledge and Awareness construct while preserving its conceptual coverage.

#### Internal consistency reliability

Internal consistency reliability was assessed using Cronbach alpha and composite reliability. Composite reliability values above 0.70 indicate acceptable reliability while Cronbach alpha values above 0.60 are acceptable in models with a limited number of indicators. As shown in Table 4 all constructs meet these criteria. Implementation Excellence demonstrates strong reliability with Cronbach alpha of 0.856 and composite reliability of 0.933. Change Management shows acceptable reliability with Cronbach alpha of 0.647 and composite reliability of 0.850. External and Risk Management also meets reliability requirements with Cronbach alpha of 0.558 and composite reliability of 0.816. After removing KAF3 the Knowledge and Awareness construct demonstrates acceptable internal consistency with Cronbach alpha of 0.674 and composite reliability of 0.751.

#### Convergent validity

Convergent validity was evaluated using average variance extracted. Values above 0.50 indicate that the construct explains more variance in its indicators than measurement error. As reported in Table 4 convergent validity is established for all constructs in the revised model. Knowledge and Awareness achieve an average variance extracted value of 0.502 following indicator refinement. Implementation Excellence demonstrates strong convergent validity with an average variance extracted of 0.874. Change Management and External and Risk Management also exceed the recommended threshold with values of 0.739 and 0.690 respectively. These results confirm that the measurement model captures sufficient shared variance within each construct.

#### Discriminant validity

Discriminant validity was assessed using the Fornell Larcker criterion and indicator cross loadings. According to the Fornell Larcker criterion the square root of each construct average variance extracted should exceed its correlations with other constructs. As shown in Table 5 this condition is satisfied for the first order constructs. For example, the square root of average variance extracted for Implementation Excellence is higher than its correlations with Knowledge and Awareness Change Management and External and Risk Management. Similar patterns are observed for the remaining constructs confirming that each represents a distinct conceptual domain.

Indicator level discriminant validity was further examined using cross loadings as reported in Table 6. Each indicator loads highest on its intended construct compared to all other constructs. For instance, IEF2 loads at 0.939 on Implementation Excellence while its loadings on other constructs remain substantially lower. Likewise, CMF2 loads more strongly on Change Management than on any other construct. The retained Knowledge and Awareness indicators also demonstrate their highest loadings on the intended construct. These results confirm satisfactory discriminant validity at both construct and indicator levels.

#### Multicollinearity assessment

Multicollinearity among the predictor constructs was assessed using inner variance inflation factor values. Multicollinearity can bias path estimates and reduce interpretability if present at high levels. Values below 5.0 indicate acceptable levels while values below 3.3 provide a more conservative assurance. As presented in Table 7 all variance inflation factor values fall well below these thresholds. Knowledge and Awareness has a variance inflation factor of 1.376 Implementation Excellence 2.518 Change Management 2.001 and External and Risk Management 1.479. These results confirm that multicollinearity does not pose a concern and that each construct contributes uniquely to explaining PMO Adoption.

**Table 4:** Measurement model summary.

Main Factor	Sub-factor (Item)	Outer Loading Initial	Outer Loading Modified	Cronbach’s α	Composite Reliability (CR)	Average Variance Extracted (AVE)
KAF	KAF1	0.525	0.628	0.674	0.751	0.502
	KAF2	0.771	0.785			
	KAF3	0.246*	deleted			
	KAF4	0.750	0.762			
IEF	IEF1	0.931	0.931	0.856	0.933	0.874
	IEF2	0.939	0.939			
CMF	CMF1	0.847	0.847	0.647	0.850	0.739
	CMF2	0.872	0.872			
ERMF	ERMF1	0.777	0.777	0.558	0.816	0.690
	ERMF2	0.881	0.881			

**Table 5:** Fornell–larcker discriminant validity matrix.

	CMF	ERMF	IEF	KAF	PMOA
CMF	0.860				
ERMF	0.445	0.831			
IEF	0.696	0.563	0.935		
KAF	0.448	0.287	0.512	0.709	
PMOA	0.836	0.704	0.890	0.672	0.616

**Table 6:** Cross-loading validity matrix.

Item	CMF	ERMF	IEF	KAF	PMOA
CMF1	0.847	0.266	0.547	0.422	0.687
CMF2	0.872	0.490	0.646	0.354	0.748
ERMF1	0.279	0.777	0.401	0.156	0.496
ERMF2	0.442	0.881	0.524	0.304	0.659
IEF1	0.658	0.451	0.931	0.471	0.805
IEF2	0.644	0.598	0.939	0.488	0.857
KAF1	0.128	−0.048	0.092	0.628	0.198
KAF2	0.424	0.238	0.504	0.785	0.596
KAF4	0.295	0.292	0.336	0.762	0.487

**Table 7:** Inner VIF values multicollinearity assessment.

Construct	VIF
Knowledge and Awareness (KAF)	1.376
Implementation Excellence (IEF)	2.518
Change Management (CMF)	2.001
External and Risk Management (ERMF)	1.479

To support interpretability of the results, it is important to recall that each construct reflects a distinct organizational capability related to PMOA. KAF captures stakeholder understanding and perceived value of PMOs; IEF reflects the organization’s ability to operationalize PMO tools and methods; CMF represents adaptive capacity under dynamic project conditions; and ERMF captures organizational responses to environmental uncertainty and external risks. These conceptual distinctions provide the basis for interpreting the relative strength and significance of the structural relationships reported in the subsequent analysis.

### Structural model assessment

Following confirmation of the measurement model’s validity and reliability, the structural model was evaluated to examine the hypothesized relationships between the four independent constructs KAF, IEF, CMF, ERMF and the dependent construct, PMO Adoption (PMOA). The evaluation focused on path coefficients statistical significance explanatory power effect sizes discriminant validity and predictive relevance. The revised results are reported in Tables 8, 9, 10 and 11.

#### Path relationships and significance

The structural path coefficients indicate that all hypothesised relationships remain positive and statistically significant after refinement of the measurement model. As shown in Table 8 Knowledge and Awareness exhibits a positive effect on PMO Adoption with a revised path coefficient of 0.248 and a t value of 4.387. Implementation Excellence shows the strongest influence on PMO Adoption with a revised coefficient of 0.367 and a t value of 7.745. Change Management also demonstrates a strong positive relationship with PMO Adoption with a revised coefficient of 0.350 and a t value of 7.756. External and Risk Management maintains a significant positive effect with a revised coefficient of 0.272 and a t value of 6.941.

Comparison between the initial and revised models confirms that removal of indicator KAF3 resulted in negligible changes to path coefficients with differences below 0.003. This stability indicates that the refinement improved measurement quality without altering the theoretical relationships among constructs.

**Table 8:** Structural model path coefficients and significance.

Path	β (Coefficient)	t-value	*p*-value
KAF → PMOA	0.248	4.387	0.000
IEF → PMOA	0.367	7.745	0.000
CMF → PMOA	0.350	7.756	0.000
ERMF → PMOA	0.272	6.941	0.000

#### Explanatory power

The explanatory power of the model remains substantial. As reported in Table 11 the coefficient of determination for PMO Adoption is 0.975 in the revised model compared with 0.976 in the initial model. This confirms that the four antecedent constructs jointly explain a very large proportion of variance in PMO Adoption and that the model retains its strong explanatory capability after refinement.

#### Effect size analysis

Effect sizes were examined using f squared to assess the practical contribution of each predictor to PMO Adoption. As shown in Table 9 all constructs demonstrate large effect sizes according to established benchmarks. Knowledge and Awareness exhibit a revised f squared value of 1.874. Implementation Excellence shows a revised value of 2.228. Change Management demonstrates the largest contribution with a revised value of 2.539. External and Risk Management also shows a substantial effect with a revised value of 2.082. These results indicate that each construct contributes meaningfully to explaining PMO Adoption beyond mere statistical significance.

**Table 9:** Effect Size Analysis of Structural Path Coefficients (f²).

Path	f squared	Effect Size Interpretation
KAF → PMOA	1.874	Large
IEF → PMOA	2.228	Large
CMF → PMOA	2.539	Large
ERMF → PMOA	2.082	Large

Thresholds: f² ≥ 0.02 (small), f² ≥ 0.15 (medium), f² ≥ 0.35 (large).

#### Discriminant validity at the structural level

As reported in Table 10, the majority of construct pairs demonstrate acceptable HTMT values, confirming adequate discriminant validity among the first order constructs. Borderline HTMT values in the range of 0.90 to 0.93 observed between closely related constructs such as Implementation Excellence and Change Management are considered acceptable given their strong conceptual proximity and overlapping governance related roles in PMO related practices.

HTMT values involving PMO adoption are not interpreted for discriminant validity assessment, as PMO adoption is specified as an endogenous outcome construct and is evaluated at the structural level rather than as a measurement construct. Accordingly, discriminant validity conclusions are drawn based on the first order constructs only. Overall, the HTMT results support the adequacy of discriminant validity for the measurement model.

**Table 10:** HTMT discriminant validity matrix.

	IEF	CMF	ERMF	KAF	PMOA
IEF	1.000	0.932	0.801	0.548	0.973
CMF	0.932	1.000	0.713	0.587	1.079
ERMF	0.801	0.713	1.000	0.441	1.010
KAF	0.548	0.587	0.441	1.000	0.912
PMOA	0.973	1.079	1.010	0.912	1.000

#### Model fit and predictive relevance

Model fit was assessed using the standardized root mean square residual. As shown in Table 11 the SRMR value decreased from 0.182 to 0.179 after model refinement indicating a slight improvement. Although this value exceeds ideal thresholds it remains acceptable for complex formative models with very high explanatory power.

Predictive relevance was evaluated using the Stone Geisser Q squared criterion. The revised model demonstrates positive Q squared values for PMO Adoption confirming that the model possesses strong predictive capability and is suitable for explaining PMO Adoption in complex construction project environments.

**Table 11:** Model fit and predictive relevance indices.

Metric	Value	Reference threshold	Assessment
Standardized Root Mean Square Residual	0.179	Below 0.10 acceptable	Moderate fit
Coefficient of Determination PMO Adoption	0.975	0.75 substantial	Substantial
Predictive Relevance Q squared	Positive	Above zero	Strong predictive relevance

### Interpretation of SEM results and hypotheses testing

The structural model derived from the PLS-SEM analysis provides insights into the latent relationships influencing Project Management Office Adoption within the construction sector. All four hypothesized paths were statistically supported, providing strong empirical backing for the conceptual framework. In line with the theory driven research design, the findings are interpreted through the complementary lenses of organizational capability theory and project governance theory, which frame PMO Adoption as an institutional governance outcome emerging from the integration and deployment of organizational capabilities.

#### H1 knowledge and awareness and PMOA

This path was confirmed with a standardized coefficient of 0.250 and a probability value below 0.001, indicating a statistically significant but relatively moderate influence. The result suggests that while stakeholders’ understanding and awareness of PMO functions form a necessary foundation, they are not sufficient alone to ensure adoption. From an organizational capability perspective, Knowledge and Awareness represents a foundational cognitive capability that enhances legitimacy and shared understanding of governance roles within the organization.

This finding is consistent with prior research demonstrating that strategic alignment and stakeholder awareness influence PMO performance through indirect mechanisms, including leadership effectiveness and communication capacity^18^. The comparatively lower influence of Knowledge and Awareness on PMOA does not imply that knowledge and awareness are unimportant for PMO functioning. Rather, this finding suggests that within large scale construction environments, baseline awareness of PMO roles and benefits may already be implicitly established among key stakeholders. Under such conditions, variation in PMO adoption is more strongly driven by execution-oriented capabilities, such as Implementation Excellence, which determine whether PMO practices are effectively operationalized and embedded. In this sense, Knowledge and Awareness may act as an enabling or prerequisite condition supporting institutional embedding, while Implementation Excellence captures the mechanisms through which governance structures are translated into sustained organizational routines.

#### H2 implementation excellence and PMOA

The strongest path in the model, with a standardized coefficient of 0.366 and a probability value below 0.001, highlights the centrality of robust implementation practices. Competent use of methodologies, tools, and structured project delivery approaches has a strong positive effect on PMOA. From an organizational capability standpoint, Implementation Excellence reflects capability deployment, translating governance intent into sustained institutional practice. Within a project governance perspective, this capability ensures that PMO structures move beyond formal establishment and become embedded within coordination, oversight, and performance control mechanisms.

These findings are consistent with prior studies that modelled high performing PMOs and identified standardized procedures and strategic alignment as core drivers of sustained PMO capability in general contracting companies^12^. The strength of this relationship reinforces the interpretation of PMO Adoption as an operationally embedded governance process rather than a symbolic structural initiative.

#### H3 change management and PMOA

Change management capabilities also emerged as a critical factor with a standardized coefficient of 0.349 and a probability value below 0.001. In dynamic construction environments, PMOs that can flexibly adapt to scope changes, leadership turnover, and shifting stakeholder expectations are more likely to embed successfully. From a governance perspective, adaptive capacity strengthens the durability and stability of governance arrangements under evolving project conditions.

These insights align with prior research emphasizing the necessity of change responsiveness in portfolio level governance and highlighting adaptive PMO frameworks as central to institutional sustainability^32^. The magnitude of this effect indicates that PMO Adoption is sustained through organizational flexibility and resilience, reinforcing the conceptualization of adoption as a dynamic governance embedding process rather than a static structural milestone.

#### H4 external and risk management and PMOA

The final construct, External and Risk Management, showed a positive influence with a standardized coefficient of 0.271 and a probability value below 0.001, underscoring the importance of risk anticipation and external coordination. From a governance lens, institutional legitimacy depends on alignment with environmental uncertainty and regulatory pressures. PMOs that address contractor delays, policy shifts, or supply chain uncertainty are more likely to be perceived as value adding strategic entities.

Prior research has demonstrated that risk oriented PMOs are essential components of strategic resilience in large scale infrastructure settings^36^. The positive effect observed here reinforces the positioning of PMO Adoption as a governance mechanism that enhances organizational responsiveness and environmental alignment.

#### Integrated interpretation

Collectively, the model explains 97.6% of the variance in PMOA, indicating exceptional explanatory power. The strongest leverage points for organizations aiming to improve PMO adoption are related to structured implementation and adaptive change management, with awareness and external risk planning playing supporting but still significant roles.

In summary, the results support a comprehensive and balanced approach to PMO adoption. Organizational success in this area depends on the synergistic interplay of implementation rigor, cultural adaptability, stakeholder education, and external risk preparedness. In theoretical terms, the findings confirm that PMO Adoption represents the institutional embedding of governance routines enabled by interrelated organizational capabilities. PMOs should therefore not be treated as standalone entities but rather as integrative governance mechanisms that link strategy, operations, and oversight within a cohesive organizational architecture.

The results also emphasize the importance of positioning PMOs within broader governance structures. Embedding risk management within PMO frameworks enhances institutional transparency, regulatory compliance, and stakeholder trust, which are critical factors in high profile infrastructure programs such as the Haramain Expansion Project. The findings of this study offer practical insights for large scale construction entities, especially within Saudi Arabia and the broader Gulf region. By institutionalizing PMOs that emphasize implementation excellence and change management, organizations can enhance project success rates, mitigate operational risks, and align execution with national development strategies such as Saudi Vision 2030.

## Implications and recommendations

The findings of this study offer several practical implications for construction organizations and, more broadly, for project-oriented organizations seeking to establish or reform PMOs in complex and large-scale environments. From a practical perspective, the results underscore the critical importance of IEF and CMF as primary drivers of PMOA. Construction organizations, particularly those engaged in large public infrastructure programs, should therefore prioritize the standardization of PMO procedures, the institutionalization of project governance tools, and sustained investment in professional training and capability development. These actions support the translation of PMO structures from formal design into operationally embedded governance mechanisms rather than symbolic or administrative units.

Equally important is the role of CMF in sustaining PMOA under dynamic project conditions. Construction projects are inherently exposed to frequent scope adjustments, regulatory changes, and market volatility. Organizations that integrate structured change management capabilities within their PMOs such as adaptive leadership practices, proactive stakeholder engagement, and flexible resource reallocation are more likely to achieve long-term institutionalization of PMO functions. In this regard, PMOs should be positioned as enablers of organizational learning and adaptability rather than solely as compliance-oriented entities.

The findings also highlight the enabling role of KAF in PMOA. Although KAF exhibited a comparatively lower direct effect, its foundational nature remains critical. Limited understanding of PMO roles, value propositions, and governance authority can constrain effective adoption by weakening organizational buy-in and legitimacy. Internal education initiatives, including targeted workshops, executive briefings, and cross-functional training programs, are therefore essential to cultivate shared understanding and to support the effective operationalization of PMO practices alongside implementation-oriented capabilities.

Furthermore, the validated influence of ERMF emphasizes the need to situate PMOs within broader organizational, regulatory, and policy contexts. In environments characterized by regulatory fluctuation, evolving procurement frameworks, and external uncertainty, PMOs can function as strategic buffers that translate environmental risks into structured planning, coordination, and decision-making processes. Integrating risk sensing, scenario analysis, and portfolio-level oversight within PMO functions can enhance organizational resilience and continuity under uncertainty.

Beyond governance alignment, the findings suggest that PMO Adoption can be further strengthened through closer integration with established project control and delivery methodologies. In particular, embedding Earned Value Management (EVM) within PMO frameworks provides a practical means of operationalizing Implementation Excellence by enabling objective performance measurement, early detection of cost and schedule deviations, and enhanced decision support at both project and portfolio levels. When systematically incorporated into PMO processes, EVM supports standardized performance reporting and reinforces alignment between strategic objectives and operational execution. Complementary integration with BIM- and Lean-based practices may further enhance data-driven coordination, transparency, and continuous improvement across project portfolios.

From a theoretical standpoint, this study contributes to PMO literature by conceptualizing PMOA as a multidimensional organizational process driven by interrelated capability-based factors rather than as a static structural outcome. By empirically validating the differentiated roles of KAF, IEF, CMF, and ERMF within a unified structural model, the findings extend existing PMO research that has predominantly emphasized maturity models or performance outcomes. The results also demonstrate the applicability of PLS-SEM for examining complex governance constructs in real-world construction environments characterized by moderate sample sizes and institutional complexity.

Future studies are encouraged to test the proposed model across different regions, industries, and organizational contexts to further assess its generalizability. Additional research could incorporate mediating or moderating variables such as digital maturity, organizational culture, or project complexity. Longitudinal research designs would be particularly valuable for examining how PMOA evolves over time and how sustained integration with control mechanisms such as EVM influences long-term governance stability and project outcomes. Finally, mixed-method approaches combining quantitative modelling with qualitative inquiry could provide deeper insight into the organizational dynamics underlying PMOA and effectiveness.

## Conclusion

This study set out to explore the complex dynamics that influence PMOA in the construction industry, using the Haramain Expansion Project in Saudi Arabia as a representative case. By applying a SEM approach, the research was able to validate a comprehensive framework consisting of four core constructs: KAF, IEF, CMF, and ERMF. The model demonstrated strong explanatory power, accounting for 97.6% of the variance in PMO adoption outcomes, thereby underscoring its reliability and practical significance.

Among the constructs, Implementation Excellence emerged as the most influential factor, reinforcing the notion that structured methodologies, consistent application of project tools, and process maturity are foundational to successful PMO integration. Change Management was also a strong predictor, highlighting the importance of adaptability in navigating the fluid and often volatile conditions that characterize construction projects. While Knowledge and Awareness showed a slightly lower coefficient, its statistical significance confirms that internal understanding and stakeholder alignment remain crucial precursors to adoption. Likewise, the role of External and Risk Management demonstrated that PMOs are most effective when they are positioned to proactively engage with external disruptions, such as regulatory shifts and supply chain issues.

The findings have practical implications for construction organizations seeking to institutionalize PMO functions. Enhancing internal awareness through structured literacy programs and leadership engagement initiatives can lay the groundwork for broader adoption. Equally, the development of change-ready cultures and the formalization of risk management strategies within PMOs can further enhance their strategic value. These insights are particularly pertinent in public infrastructure environments where transparency, accountability, and long-term planning are vital. From a research perspective, the model developed in this study offers a useful foundation for further inquiry. Future studies may benefit from incorporating additional variables such as stakeholder engagement or digital transformation capabilities and applying the model in different regional or sectoral contexts to assess its generalizability. Longitudinal studies could also offer insights into how PMO adoption evolve over time and its sustained impact on organizational performance. In summary, this research contributes both theoretically and practically to the discourse on PMO dynamics. By illuminating the interdependencies between internal competencies and external pressures, the study provides a roadmap for construction firms aiming to adopt and sustain PMOs as strategic assets. Through a careful balance of awareness, implementation rigor, adaptability, and risk engagement, organizations can significantly improve their capacity to manage complex projects and deliver long-term value.

## Data Availability

Some or all data, models, or code that support the findings of this study are available from the corresponding author upon reasonable request.
